# Direct and accurate measurement of size dependent wetting behaviors for sessile water droplets

**DOI:** 10.1038/srep18150

**Published:** 2015-12-14

**Authors:** Jimin Park, Hyung-Seop Han, Yu-Chan Kim, Jae-Pyeong Ahn, Myoung-Ryul Ok, Kyung Eun Lee, Jee-Wook Lee, Pil-Ryung Cha, Hyun-Kwang Seok, Hojeong Jeon

**Affiliations:** 1 Center for Biomaterials, Korea Institute of Science & Technology, Seoul, 02792, South Korea; 2 Korea University of Science and Technology, Daejeon, 34113, South Korea; 3 Advanced Analysis Center, Korea Institute of Science & Technology, Seoul, 02792, South Korea; 4 School of Advanced Materials Engineering, Kookmin University, Seoul, 02707, South Korea

## Abstract

The size-dependent wettability of sessile water droplets is an important matter in wetting science. Although extensive studies have explored this problem, it has been difficult to obtain empirical data for microscale sessile droplets at a wide range of diameters because of the flaws resulting from evaporation and insufficient imaging resolution. Herein, we present the size-dependent quantitative change of wettability by directly visualizing the three phase interfaces of droplets using a cryogenic-focused ion beam milling and SEM-imaging technique. With the fundamental understanding of the formation pathway, evaporation, freezing, and contact angle hysteresis for sessile droplets, microdroplets with diameters spanning more than three orders of magnitude on various metal substrates were examined. Wetting nature can gradually change from hydrophobic at the hundreds-of-microns scale to super-hydrophobic at the sub-μm scale, and a nonlinear relationship between the cosine of the contact angle and contact line curvature in microscale water droplets was demonstrated. We also showed that the wettability could be further tuned in a size-dependent manner by introducing regular heterogeneities to the substrate.

Understanding the wetting behavior of nano- and micro-sessile droplets has been a challenge in the discovery of fundamental biological and physical phenomena and the development of numerous electrical and biomedical devices^1^,^2^,^3^,^4^,^5^,^6^,^7^,^8^,^9^,^10^. Among the various wetting phenomena, the size-dependent wetting physics of droplets has remained one of the most important issues because this phenomenon can provide a foundation for designing novel micro-/nanofluidics, micro-/nanofabrication, and thin film coating techniques, which require sophisticated handling of sessile droplets of controlled size^11^,^12^,^13^,^14^,^15^,^16^,^17^,^18^,^19^. Previously, this phenomenon was widely investigated by combining imaging tools, such as atomic force microscopy (AFM)^20^,^21^,^22^,^23^,^24^,^25^, environmental scanning electron microscopy (ESEM)^26^,^27^,^28^, and cryogenic scanning electron microscopy (cryo-SEM)^29^,^30^,^31^, and theoretical modeling^12^,^21^,^32^,^33^,^34^. However, previous studies have mainly focused on the wettability of nucleated droplets in a closed system^25^,^28^,^29^. Therefore, the underlying principle of wetting nano- and micro-sessile droplets remains un-resolved. Moreover, although the droplets are initially formed by a sessile mechanism, inherently fast evaporation and condensation kinetics have impeded their accurate analysis^7^,^35^,^36^,^37^.

Previous studies have observed the different physical statuses of sessile water droplets and nucleated droplets. Sessile water droplets were deposited under a thermodynamically non-equilibrium condition, whereas nucleated droplets formed under an equilibrium condition^7^,^36^,^38^,^39^,^40^. Although there was no significant macroscopic difference in the wettability of sessile and nucleated droplets, the non-equilibrium effect cannot be neglected for microscopic sessile droplets^36^,^39^. In particular, the dynamic evaporation of sessile droplets is regarded as the major cause of deviation from the thermodynamic state^36^. Although many ESEM or AFM studies have examined the wettability of thermodynamically stable droplets formed by condensation or evaporation with delicate control of the surrounding temperature and pressure^41^,^42^,^43^, less is known about sessile droplets because of their dynamic evaporation. In this regard, a system that can directly observe sessile water droplets without morphological and volumetric changes during analysis can address the science of microscale sessile water droplets and decrease the non-equilibrium effect. In this regard, cryogenic-focused ion beam (FIB) milling and the SEM imaging technique (cryo-FIB/SEM) have an advantage in decreasing the evaporation effect using a rapid freezing rate (20,000 °C/s)^29^.

Herein, we have selected cryo-FIB/SEM as a model system to investigate the size-dependent wetting behavior of microscale sessile water droplets. Directly visualizing the three-phase interface, the wettability of sub-1 to 200 μm droplets was accurately investigated with a decreased evaporation effect under a cryogenic temperature. We showed that the contact angles of the microscale sessile droplets significantly changed in a size-dependent manner, and the super-hydrophobic nature of droplets that are only a few micrometers in diameter was directly imaged for the first time. These results were discussed with several well-established theoretical models. Furthermore, by introducing a nanopattern to change substrate topology, we demonstrated that the degree of anisotropic wetting depends on droplet size.

## Results

### Visualization of microscale sessile droplets

**Figure 1:**
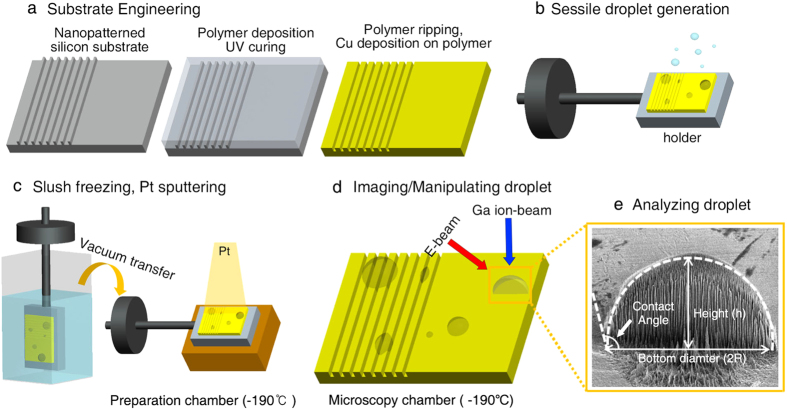
Schematic of the droplet analysis using the cryo-FIB/SEM system. (**a**) Substrate engineering by Cu deposition on the patterned/non-patterned polymer substrate. (**b**) Generation of the sessile droplet on the engineered Cu substrate. (**c**) Rapid slush freezing of the droplet and consequent Pt sputtering under a cryogenic temperature. (**d**) Electron and gallium ion beam integrated milling and imaging of the droplet under a cryogenic temperature. (**e**) Wettability analysis of the FIB-milled droplets.

Sessile water droplets were prepared using a simple spray method onto metal substrates. The metal substrates were fabricated by sputtering 50-nm thick Cu film onto two types of perfluoropolyether (PFPE) polymer substrate: one with a flat morphology and another with a simple grooved pattern (Fig. 1a). Cu was selected for its excellent thermal conductivity, ensuring the fast cooling of the droplet^44^. The Cu sputtered substrates were mounted onto a Cu metal stub of cryo-SEM and connected to a transfer holder. De-ionized water droplets were then sprayed onto the metal substrate at room temperature before rapid plunge freezing in liquid nitrogen slush (Fig. 1b). After the samples were submerged into liquid nitrogen slush for approximately 1 s, we directly transported them into a high-vacuum sample preparation chamber to minimize the possible formation of condensed droplets. The samples were then coated with a conductive platinum layer (~5 nm) under cryogenic temperature (−190 °C) (Fig. 1c). Finally, the platinum-coated samples were transferred to a microscope stage where the temperature was maintained below −190 °C.

**Figure 2:**
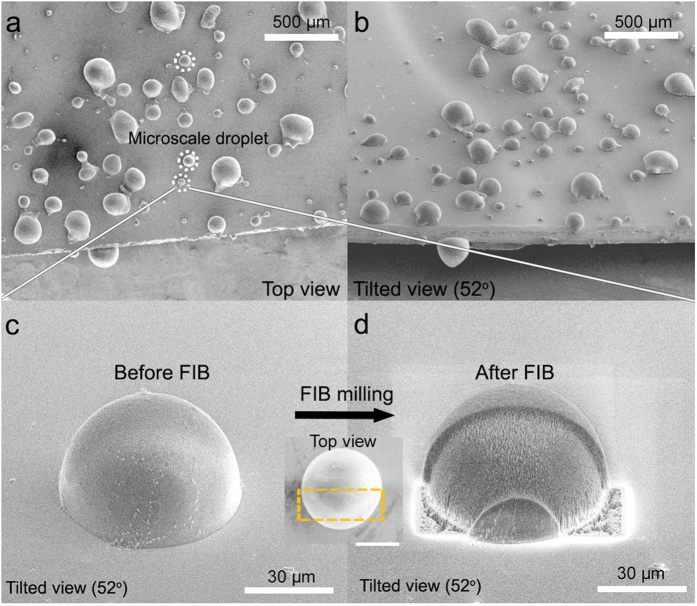
Cryo-SEM images of sessile water droplets. (**a**) Top and (**b**) tilted views of the droplets with various diameters on the Cu substrate. Typical tens of micrometers scale is indicated by white circles. (**a**) Magnified images of the 62.4-μm diameter droplets before (**c**: tilted view, **c** inset: top view) and after (**d**) FIB milling of selected area (inside the orange dotted line in **c** inset) under a cryogenic temperature.

As shown in Fig. 2a,b, various sizes of sessile droplets, from sub-micrometer sizes to hundreds of micrometers, can be successfully formed using this method. We observed that droplets with a diameter larger than tens of micrometers exhibited a hemispherical shape from the top-view and tilted cryo-SEM images (Fig. 2a,b). Any significant morphological deformations that could have resulted from the crystallization of the droplets were not observed. This result is consistent with a previous study that showed that nano- to microscale water droplets can be vitrified with negligible volumetric/morphological change via nitrogen slush plunge freezing^29^. Compared to conventional freezing procedures performed at ~ −20 °C, the freezing rate using slush-plunge liquid nitrogen in our study was considerably faster with a value of ~20,000 °C/s^29^,^45^,^46^,^47^. Using the slush plunge freezing method, microscale water droplets can be vitrified within a few microseconds, which is notably faster than the evaporation or condensation processes that can result in morphological changes to the droplets with the timescale of milliseconds^29^. It is worthwhile to note that the conventional freezing process performed at ~−20 °C can produce droplet evaporation or re-condensation, which may prevent an accurate analysis for the wettability of the sessile droplets^47^.

In some cases, considerably large droplets, more than 300 μm, were irregular circles with minor deformations (Fig. 2a,b). However, it should be noted that their sizes were larger than the typical droplet range for plunge frozen specimens (less than ~300 μm)^48^. Consequently, a phase transition can occur during the cryo-stabilization procedure^48^. These types of artifacts can also be observed in slush plunge freezing droplets in previous studies^29^. Thus, we excluded the analysis of these irregularly shaped, hundreds-of-micrometers scale droplets because of their possible morphological change during the cryo-stabilization step. Moreover, when we employed control experiments, in which no spraying was performed before the nitrogen slush plunge freezing, nearly no water droplets were observed on the substrate. If the observed droplets were formed via the condensed mechanism, some water droplets would have been detected in the control experiments. This control experiment implies that the droplets studied in our experiments were predominantly formed by the sessile mechanism.

**Figure 3:**
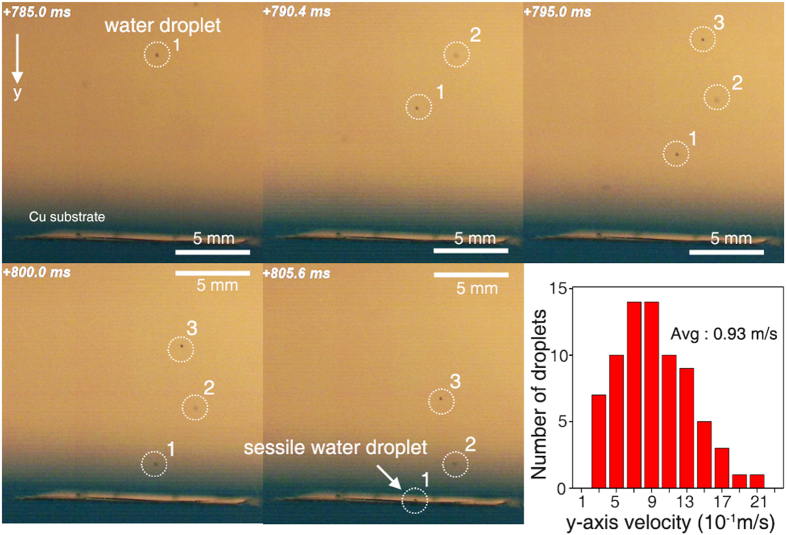
Snapshots of sessile droplet formation. Formation of the sessile water droplets using the spraying method as recorded by a high-speed camera at 10,000 frames per second. The graph displays the distribution of droplet velocities along the y-axis. The average droplet velocity is 0.93 m/s.

The detailed fluidic mechanics of the sprayed droplets are considered below. Using a high-speed camera (10,000 frames per second), we initially measured the velocity of the sprayed droplets when they were placed on the Cu substrate (Fig. 3, Supplementary Movie 1). With the theoretical density, surface tension of the water and average droplet velocity, we calculated the Weber number and Reynolds number^49^,^50^. Because the sizes of the observed sessile droplets were less than 300 μm, the upper limit of the Weber number and Reynolds number of the droplets in our system were 3.6 and 280, respectively. According to previous studies, in the experimental conditions where the Weber and Reynolds numbers were less than 4 and 400, respectively, the impact evolution of the droplets exhibited sticky vibrating water types without a splash impact^49^,^50^. Because of their small size, it was difficult to observe the exact impact evolution of sessile droplets using the high-speed camera; however, an apparent splash impact was not observed (Supplementary Fig. 1).

Moreover, Rioboo *et al.* reported that when dimensionless time (t*), which is defined by vt/R (Here, v and R indicate the velocity and radius of the droplets), becomes more than 1,000, the deposited sessile water droplets retain their equilibrium shape, or so-called pure wetting phase status^51^. In our case, the corresponding times to achieve a pure wetting phase status were 42 and 1.1 ms for 80- and 2-μm diameter droplets, respectively. Considering the time interval between creating and freezing the droplets was approximately 0.5 s, we hypothesized that the droplets in our study were in the pure wetting phase status.

Additionally, because previous studies have shown that the evaporation rates of few micrometer-sized droplets were high^35^,^36^, we calculated the expected mass loss for the droplets in our study during the preparation process. We initially explored whether the droplets followed a constant contact diameter mode or constant contact angle mode^52^,^53^,^54^,^55^. Contact angle hysteresis tests showed that the droplets followed a nearly constant contact angle mode with a small difference between the advancing and receding contact angles (Supplementary Table 1)^52^,^53^,^54^. The evaporation rates of the droplets can be considered as follows^35^,^55^:


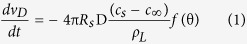


in which D is the diffusion coefficient (cm^2^/s); *C*_*S*_ and *C*_∞_ are the vapor concentrations at the sphere’s surface and at an infinite distance (g/cm^3^), respectively; *ρ*_*L*_ is the density of the droplet (g/cm^3^); and 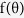 is a function of the contact angle of the spherical cap. For the constant contact angle evaporation mode, the change in the contact angle is negligible compared to that for the contact radius, and Erbil *et al.* suggests that the contact radius can be calculated as a function of time^55^:


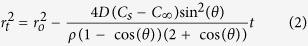


Using the diffusion coefficient of water (0.24 cm^2^ s^−1^), the measured contact angle described in the later section of this manuscript, and time intervals between creating and freezing the droplets (<0.5 s), we can calculate the change in contact radius before freezing. The concentration of vapor at the droplet sphere surface was calculated according to the Kelvin equation^36^:


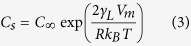


in which R is the curvature radius of the drop; V_m_ is the molecular volume in the liquid phase; and k_B_, *γ*, and T are Boltzmann’s constant, surface tension, and temperature, respectively.

Based on these equations, the change in radius of 2-μm diameter droplets (*r*_*o*_ = 1 μm) with a contact angle of ~130^o^ was calculated, indicating that approximately 10.9% of the mass in 2-μm-diameter droplets evaporated before freezing. For typical 80-μm-diameter droplets with a contact angle of ~90^o^, the mass loss before freezing was expected to be approximately zero. Although a portion of some micrometer-sized droplets was expected to evaporate during the preparation process, it should be noted that slush-plunge freezing requires a relatively shorter preparation time and enabled us to explore the wettability of the droplets with less evaporation effect compared to common AFM analysis, which requires at least approximately 15–30 min to obtain an image^7^.

### Manipulating the droplets for analyzing the contact angle

The droplet contact angles were measured using a top view and cross-sectional images (Figs 1d,e and 2c,d). The droplet diameters were measured from top view images when the z-axis of the droplets was parallel to the electron-beam source. To measure droplet height, the microscope stage was tilted by 52° to make the z-axis of the droplets parallel to the gallium ion-beam source. After removing one-half of the droplets, droplet height was measured with a tilted-view image. Because the z-axis of the droplets was not parallel to the electron-beam source after stage tilting, the measured height (H) was corrected using the following equations to obtain the actual height (h) of the droplets (Supplementary Fig. 2):


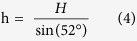


A high ion current value can damage the topmost surface of the droplets during the milling process. Therefore, to minimize potential damage, we used a small current value of 1 nA, which was similar to the previously reported current value for milling nano- and microscale droplets^29^. Additionally, we did not perform an additional Pt coating before the FIB process to exclude the heat effect from such organometallic gas exposure, which consequently deforms droplet morphology^29^.

Cryo-SEM images of the 62.4-μm droplet before and after the FIB-milling process are shown in Fig. 2c,d. The radius (R) of the droplets was carefully measured from a top view image before FIB-milling, assuming the top view morphology of the droplet was a perfect circle (Figs 1e and 2c). Based on the low contact angle hysteresis for our metal substrates, we think that our experimental results followed a weak heterogeneities case as discussed by Joanny and De Gennes^54^. Checoo *et al.* reported that droplets with low contact angle hysteresis (1–2^o^) (weak heterogeneity case) maintain an approximately spherical shape^21^. Indeed, tens of micrometers droplets have a near-circular shape from the top-down perspective (Fig. 2c inset). Moreover, the contact angle of microscale water droplets with considerably small Bond numbers (≪0.5) can be perceived as having a spherical cap shape^56^. Considering that even droplets with a large Bond number on a homogenous surface could be perceived as spherical, we believe that our microscale water droplets with small contact angle hysteresis and Bond number can be deduced as spherical shape. After a portion of the droplets was removed by FIB-milling, the heights (h) of each droplet were carefully measured (Figs 1e and 2d). Some line patterns were formed at the cross-sectional region after FIB milling as shown in Figs 1e and 2d. However, these patterns were observed in the typical FIB procedure, and more importantly, they did not significantly affect the radius or height of the droplets^29^,^57^. Finally, the contact angles of the droplets were calculated by the following equations, assuming that the cross-sectional images of the droplets were a component of the perfect circle:


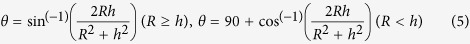


### Size-dependent wettability of the droplets

Using equation (5), we observed that the accurate contact angle of the 62.4-μm diameter droplet was 98.2°. Using the identical analytical method for other droplets with different sizes, we observed that the contact angle of the droplets gradually increased as their size decreased. For example, the contact angle of a 12.6-μm diameter droplet was 115.8°, which is approximately 30° higher than that of a 253-μm diameter droplet (85.3°). The correlation between the contact angle and size of the droplets will be discussed in the latter portion of this manuscript.

**Figure 4:**
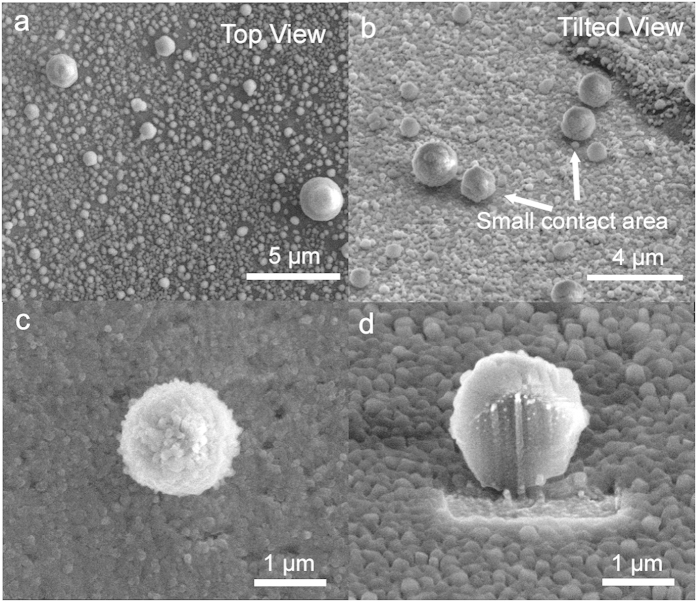
Spherical water droplets. Cryo-SEM images of sub-5-μm diameter water droplets on a Cu substrate (**a**: top view, **b**: tilted view). The tilted image (**b**) obviously shows that these droplets have a negligible contact area with the solid substrate (white arrows). Magnified images of 1.3-μm diameter droplets before (**c**) and after (**d**) FIB milling.

Notably, we observed that the contact angle of the droplets increased drastically when the droplet diameter was less than 5 μm. Top and tilted images of these droplets showed a nearly spherical shape with a small contact area on the solid substrate (Fig. 4a,b). Such a wetting phenomenon is slightly different compared to that for droplets occurring at the tens of micrometers scale that have hemispherical morphology and a broad contact region on the solid substrate. The magnified image of a 1.3-μm-diameter droplet is shown in Fig. 4c. The cross-section of the droplet was a circle with a small contact area on the metal substrate (Fig. 4d). Based on equation (5), we can determine that the contact angle values were more than 130°. In Fig. 4, we think that ultra-small granules on the substrate may be unavoidably formed nucleated droplets from vapors in the air during freezing and sample-transfer^58^. Indeed, when we tentatively increased the humidity during freezing and sample-transfer, we observed that many similar small granules formed on the substrate (Supplementary Fig. 3). To verify whether these small particles were water droplets, we performed an energy-dispersive X-ray spectroscopy (EDS) elemental analysis. The acquired spectra showed that the particles were mainly composed of oxygen as expected, thus confirming that the spherical particles were water droplets (Supplementary Fig. 4). Additionally, when we intentionally raised the temperature of the metal stub on the microscope stage from −190 to 20 °C, the size of the particles gradually decreased because of sublimation. At approximately −20 °C, most particles disappeared (Supplementary Movie 2). A similar spherical droplet was also observed when we used aluminum and silver substrates (Supplementary Fig. 5).

**Figure 5:**
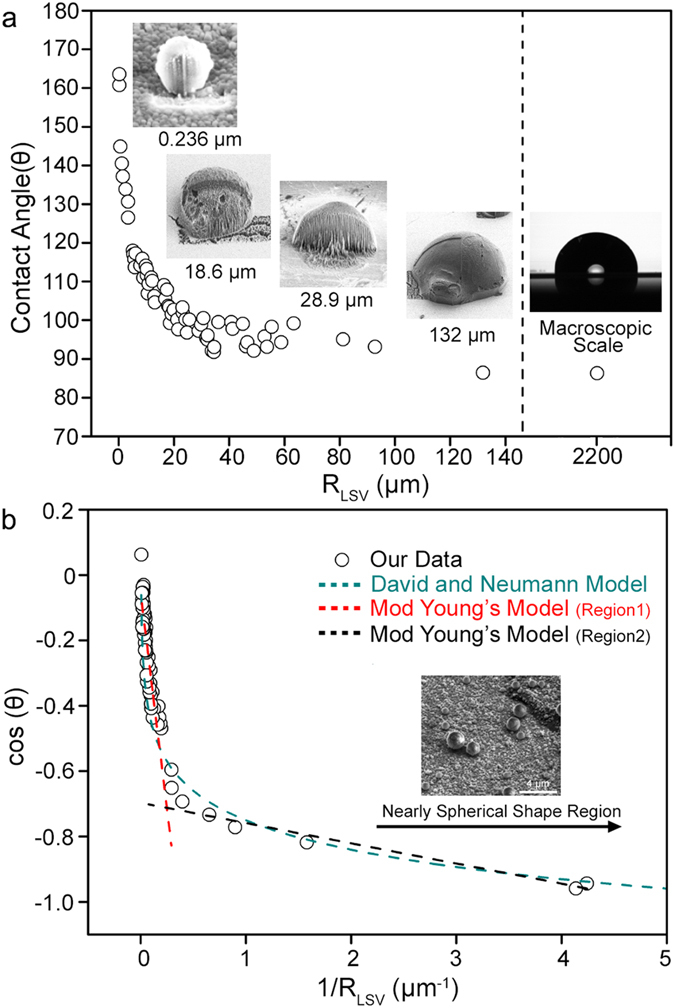
Size-dependent droplet wetting. (**a**) The contact angle (*θ*) value of the droplets as a function of R_LSV_. Representative images of four droplets with different R_LSV_ values are displayed in inset (**a**). (**b**) Plot of cos(*θ*) versus 1/R_LSV_ of the droplets fitted with various theoretical models.

To understand the correlation between droplet size and wetting behaviors in a systematic and quantitative manner, we plotted several diagrams based on various values of the droplets, including R_LSV_, contact angle, and curvature. Here, R_LSV_ refers to the contact line radius where the three phases intersect. Different wetting behaviors that depend on droplet size could be observed when we plotted the contact angle of the droplets as a function of R_LSV_ (Fig. 5a). With increasing R_LSV_ from 0.236 to 132 μm, the contact angle value decreased approximately 75.4° from 160.7° to 85.3°. Based on the resolution of cryo-SEM (3.0 nm), the errors resulted from the imaging resolution were expected as approximately ± 0.3° and ± 0.05° for the sub-1-μm droplet and 200-μm droplet, respectively. Since it is difficult to form the droplets with identical diameters, we further calculated the errors by comparing the contact angle values of the droplets with a similar contact angle radius. Specifically, the following droplet contact angles were observed at specific R_LSV_ ranges: 116.3 ± 1.5° (5.6 ± 0.4μm), 105.9 ± 2.2°(17.4 ± 0.7μm), and 97.4 ± 2.1°(30.6 ± 1.2μm) (each error bar presents five sets of image data at the specific R_LSV_ ranges). The contact angle value varied dramatically with an R_LSV_ value of less than 5 μm, whereas R_LSV_ values of more than 80 μm approached a saturated value of 58.3° with minimal change. For droplets greater than 130 μm, the contact angle was nearly identical with that of millimeter-sized droplets (86.3°) as measured by a conventional contact angle measurement device. This result implies that the effect of size on the wetting property was minimal at this scale. The inset images in Fig. 5a show representative examples displaying an increase in the contact angle with a decreasing droplet size.

**Figure 6:**
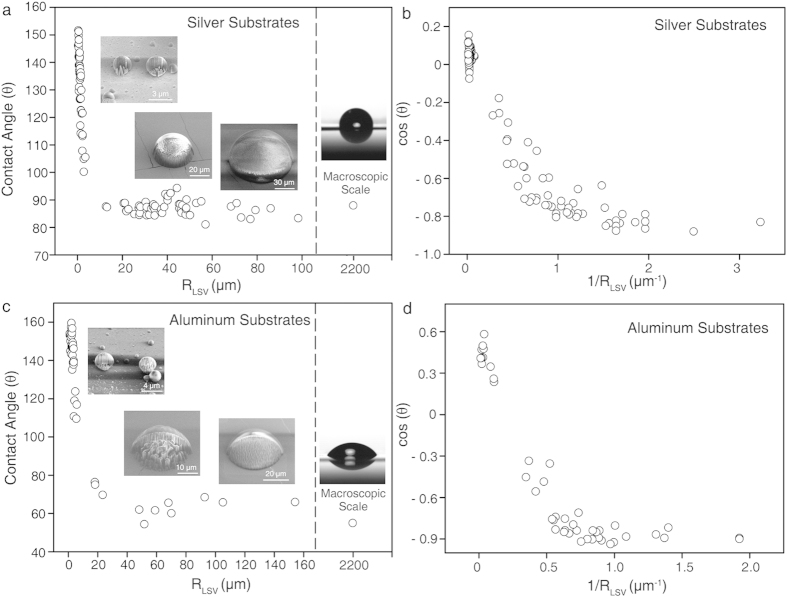
Size-dependent wetting of droplets on the Ag and Al substrates. (**a**,**c**) The contact angle (*θ*) of the droplets as a function of R_LSV_ on Ag and Al. Cryo-SEM images of sessile water droplets (tilted-view) and an optical image of the macroscopic droplet (side-view) are displayed in the **a** and **c** insets. (**b**,**d**) Plot of cos(θ) versus 1/R_LSV_ of the droplets on Ag and Al, respectively.

Additionally, we explored the wettability change of sessile droplets with size on two different substrates, Al and Ag, which have excellent thermal conductivities that are comparable to Cu. Using the identical preparation method to create sessile droplets on a Cu substrate, a wide range of diameters droplets were formed on the different metal substrates. After the rapid-freezing process, each droplet was analyzed with the cryo-SEM/FIB technique (Fig. 6).

Initially, the change in droplet contact angle was explored as a function of the three-phase contact line radius (R_LSV_). Notably, similar to our observation for Cu, a sharp increase in the contact angle was observed with a decrease in radius (Fig. 6a,c). Moreover, we observed that the contact angle became saturated as radius increased toward that of the macroscopic droplet, further suggesting that our technique can be complementary to the conventional contact angle meter (Fig. 6a,c).

### Size-dependent anisotropic wetting on grooved substrates

**Figure 7:**
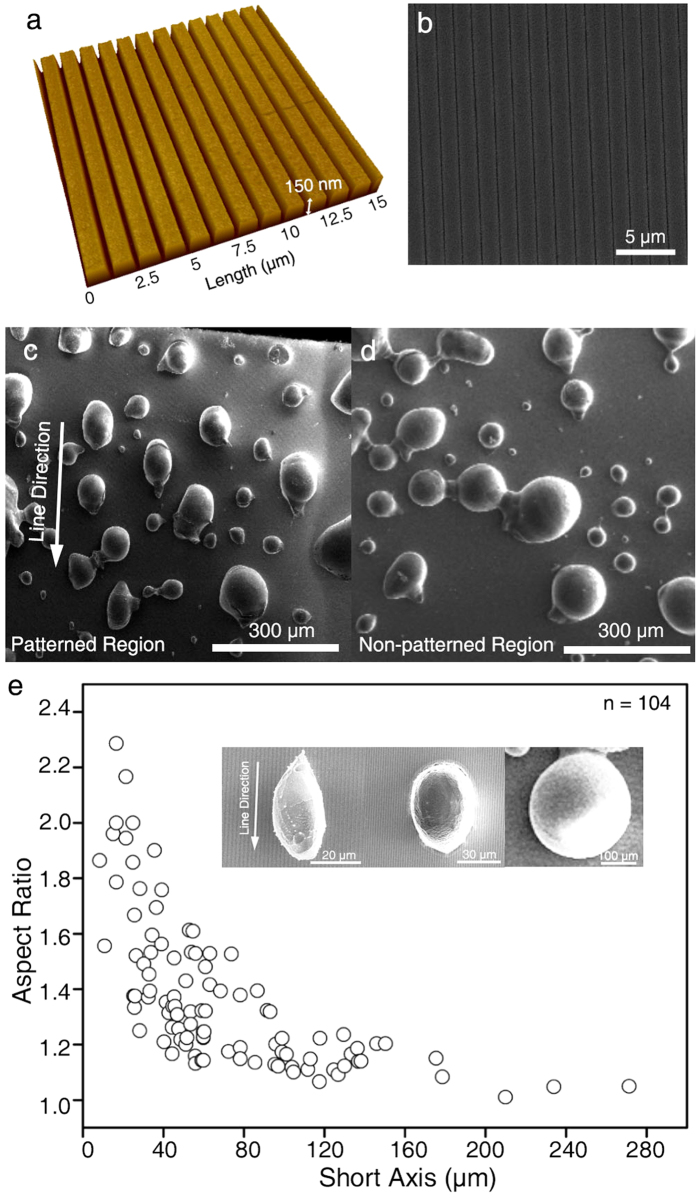
Anisotropic wetting on grooved substrates. AFM (**a**) and SEM (**b**) images of the nano-patterned Cu substrate. Cryo-SEM images of water droplets on the patterned Cu substrate (**c**) and non-patterned substrate (**d**). The pattern direction is displayed by a white arrow (**c**). The aspect ratios of the droplets are a function of their diameter (**e**). Three representative droplets are shown in the inset. The aspect ratio of the droplets gradually increases with decreasing droplet size.

A nano-patterned Cu substrate with high heterogeneity was prepared as a platform system to better understand the size-dependent wetting behavior. We intentionally used simple grooves (150 nm deep, 400 nm wide, and 1 μm in pitch) with a rectangular cross-section to identify the effect of heterogeneity on wetting as a function of the degree of anisotropic wetting (Fig. 7a). The sessile droplets were created using the same method as employed for the flat Cu substrate. The morphology of the pattern was maintained during the imaging process under a cryogenic temperature (Fig. 7b).

We showed that the sessile water droplets spread along the grooves (line patterns) with an elliptical shape instead of the nearly perfect circle shape maintained on non-patterned regions (Fig. 7c,d). To systematically understand size-dependent anisotropic wetting on this pattern, we plotted the aspect ratio of the droplets as a function of their size (Fig. 7e). The aspect ratio has been used to analyze the degree of the anisotropic wetting of droplets and the effect of a grooved pattern on the wettability of droplets in previous studies^59^,^60^,^61^,^62^. The aspect ratio was measured by dividing the length of the droplets along the line pattern by the length perpendicular to the pattern. The aspect ratio deviated from 1.0 when the short axis of the droplet was less than 150 μm, and the ratio increased as droplet size decreased. For example, the aspect ratio of a 20-μm droplet was approximately 2.0. When droplet size was more than 200 μm, the aspect ratio of the droplets was approximately 1.0, indicating that the effect of the nano-pattern was no more dominant in this region. The representative images in the Fig. 7e inset obviously show that the degree of anisotropy of the droplets increases as size decreases. Indeed, previous reports showed that contact angles become anisotropic using several surfaces with different groove widths and heights^59^,^62^. Although underlying mechanism is not well-understood, one possible explanation for this phenomenon is that the droplet may be in a receding state because of the relatively high vaporization rate during the freezing step.

## Discussion

We investigated the possible reasons for the change in droplet contact angle with size. For the initial step in the analysis, we considered the influence of contact line tension (CLT), which originates from an imbalance in regional forces where the three phases intersect^11^,^12^,^13^,^63^,^64^. Previous studies have shown that the contact angle values can change because of CLT on micro- or nanoscale droplets, and Young’s equation was modified into the following equation^12^,^17^,^63^,^64^:


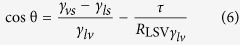


in which *γ* and *τ* are surface tension and line tension, respectively. We plotted the correlation between cos(θ) and 1/R_LSV_ of the sessile droplets in our study (Fig. 5b). However, we did not determine the universal linear equation that covered all droplet sizes in our system. Instead, we re-plotted our data by dividing it into two regions: one more than 5 μm of R_LSV_ and another less than 5 μm of R_LSV_. Linear trends were observed with a negative slope value of -2.6×10^−6^ m and −6.2 × 10^−8^ in the larger and smaller regions, respectively. Based on the surface tension of water (0.072 J/m^2^)^65^, the line tension values were calculated as 1.9 × 10^−7^ *N* and 4.5 × 10^×9^ *N*. These values were notably higher than the theoretically expected value (~10^−12^ *N*), implying that CLT may not be the only factor determining wetting behavior^21^,^34^,^66^,^67^,^68^,^69^,^70^. Additionally, physical evidence for the discrete transition of the line tension value at a specific droplet size remains unknown. Moreover, nonlinear dependency between the two factors was also observed for the Al and Ag substrates and was similar to our observation for Cu films (Fig. 6b,d).

To investigate another possible correlation between cos(θ) and R_LSV_, we verified a nonlinear model, such as the David and Neumann model, which describes the logarithmic relationship between two factors based on empirical data^71^. This model experimentally observed that the line tension value (σ) was roughly proportional to droplet radius (R), and a logarithmic relationship was derived based on these experimental results. Additionally, the authors noted that long-range forces, such as intermolecular forces in the presence of dipole charges, can lead to the linear dependence of σ and R, thus suggesting the physical significance of these long-range forces on wettings^71^,^72^. We observed that fitting cos(θ) versus the curvature to a logarithm was consistent with our data, producing an equation of cos(θ) = −0.20 ln (1/R) – 0.85 (Fig. 5b). Within a narrow range of the plot, the logarithmic equation appeared as a straight line, implying that the logarithmic relationship was more accordant with our empirical data than a linear relationship. However, as R goes to infinity, the cos(θ) value increased dramatically and became larger than one, which was its theoretical limit. This value was contrary to our experimental results, which showed a saturated contact angle with increasing droplet size.

We then focused on substrate heterogeneity, which can influence the wetting behaviors of microscale water droplets^21^,^67^. Checco *et al.* experimentally presented nonlinear dependency in condensed alkane droplets and observed the dominant role of substrate heterogeneity on their wetting over CLT^21^. Additionally, Neumann and colleagues theoretically showed that even a small heterogeneity of the solid surface, such as corrugations of the three-phase contact line, could result in nonlinear dependency^67^, which is in agreement with our experimental results. For the Cu substrate, four independently prepared samples were analyzed and the roughness value of each sample exhibited a similar value of approximately 8 nm. The roughness values of the Ag and Al substrates were approximately 1.0 and 4.5 nm, respectively (Supplementary Fig. 6). These results imply the possible effect of substrate heterogeneity on the wetting of microscale droplets in our system.

Although it remains unclear how surface heterogeneity induces size-dependent wettability, we believe that these systemic evaluations for the wetting behaviors of volatile, sessile droplets with direct visualization can provide a valuable experimental basis to verify the validity of the established or newly derived theoretical models. Moreover, we are currently in the process of integrating the above-mentioned factors to further understand the wetting phenomena of sessile water droplets.

In conclusion, we directly demonstrated the size-dependent wetting behavior of sessile droplets with a new level of accuracy using the cryo-FIB/SEM technique. A wide range of droplets was observed, and dramatic changes in wettability were dependent on droplet size. We observed that droplet wettability can be super-hydrophobic when droplet size decreased to approximately 5 μm, and the nonlinear dependency between the cosine of the contact angle and contact line curvature was identified in microscale water droplets. Additionally, the size-dependent variation of droplet wettability was demonstrated on non-planar substrates by exploring changes in the degree of anisotropic wetting. We strongly believe that these results mark an important step toward the profound understanding of wetting behaviors of sessile droplets. Moreover, we believe that this systematic approach can be extended beyond this particular size-dependent wetting phenomenon and have a broader impact on other wetting phenomena in nature, such as surface dependent cell movement, cell/protein adhesion, and anti-biofouling events.

## Methods

### Material fabrication and characterization

Patterned polymer substrates were prepared according to previous studies^73^. Photo-curable PFPE solution was poured onto a patterned Si-wafer developed by soft-lithography. After 3 min of UV curing, polymerized PFPE films were carefully detached from the Si master. Before metal deposition onto the films, the films were thoroughly rinsed with ethanol to remove uncross-linked residues. The flat polymer substrates were prepared using a non-patterned, flat Si wafer. Metal deposition was then performed on the flat/patterned substrates at a chamber pressure of 2 × 10^−6^ Torr for 10 min using a magnetron sputter deposition machine (Korea vacuum system, Korea). AFM images of the metal deposited films were obtained using XE-100 park systems (Korea) in non-contact mode. The operating conditions for the AFM were 0.4 Hz of the scan rate.

### Cryo-FIB/SEM imaging

The cryo-FIB/SEM experiments were performed using Quanta 3D FEG (FEI, Netherland) with an Alto 2500 cryo-transfer system (Gatan, UK). The Cu-deposited polymer substrates were attached to a copper stub using carbon tape. After spraying the sessile droplets onto the substrates, the substrates were rapidly submerged into liquid nitrogen slush within 0.5 s. After approximately 1 s, the freezing chamber was evacuated. The samples were then directly transferred into a preparation chamber, which was pre-evacuated to a pressure of 10^−5^ mbar at approximately −190 °C. Metal deposition was performed by plasma sputtering with a 3-mA current for 60 s. The metal-coated samples were transferred into a microscope chamber, which was also pre-evacuated to a pressure of 10^−5^ mbar and precooled to a temperature of −190 °C. The cryo-SEM image was acquired with a 5-keV electron beam of energy and an electron current of 11.8 pA. FIB milling was performed with a 30-keV gallium ion beam and an ion current of 1 nA. The electron beam energy was increased to 15 keV to collect the EDS spectra.

### High-speed camera and contact angle hysteresis analysis

The dynamics of sessile droplet formation was recorded using a high-speed camera (Fastcam SA1, Photron) with a recording rate of 10,000 frames per second. The sessile droplet was formed with the spraying method, which was also used for our cryo-SEM/FIB analysis. Contact angle hysteresis was measured using a contact angle meter (FemtoFab, SmartDrop SD110TEZ) with the tilting plate method. The volume of a drop was fixed at 2 μl for the measurements.

## Additional Information

**How to cite this article**: Park, J. *et al.* Direct and accurate measurement of size dependent wetting behaviors for sessile water droplets. *Sci. Rep.* **5**, 18150; doi: 10.1038/srep18150 (2015).

## Supplementary Material

Supplementary Video 1

Supplementary Video 2

Supplementary Information
