# Erratum: Direct and accurate measurement of size dependent wetting behaviors for sessile water droplets

**DOI:** 10.1038/srep46836

**Published:** 2017-06-26

**Authors:** Jimin Park, Hyung-Seop Han, Yu-Chan Kim, Jae-Pyeong Ahn, Myoung-Ryul Ok, Kyung Eun Lee, Jee-Wook Lee, Pil-Ryung Cha, Hyun-Kwang Seok, Hojeong Jeon

Scientific Reports 5: Article number: 18150; 10.1038/srep18150 published online: 12 14 2015; updated: 06 26 2017.

This Article contains errors. In the ‘Results’ section, under the subheading ‘Size-dependent wettability of the droplets’.

“The contact angle value varied dramatically with an R_LSV_ value of less than 5 μm, whereas R_LSV_ values of more than 80 μm approached a saturated value of 58.3° with minimal change.”

should read:

“The contact angle value varied dramatically with an R_LSV_ value of less than 5 μm, whereas R_LSV_ values of more than 80 μm approached a saturated value of 85.3° with minimal change.”

