# Supplementary material for: Direct and accurate measurement of size dependent wetting behaviors for sessile water droplets
Source: Sci Rep. 2015 Dec 14;5:18150. doi: 10.1038/srep18150 (PMC4677463; doi:10.1038/srep18150)
Supplement: Supplementary Information [file srep18150-s3.pdf]

Supplementary Information

Direct and accurate measurement of size dependent wetting behaviors for sessile water droplets

Authors: Jimin Park[1], Hyung-Seop Han[1], Yu-Chan Kim[1,2], Jae-Pyeong Ahn[2,3], Myoung-Ryul Ok[1], Kyung Eun Lee[3], Jee-Wook Lee[4], Pil-Ryung Cha[4], Hyun-Kwang Seok[1,2] & Hojeong Jeon[1,2]*

Affiliations:

[1] Center for Biomaterials, Korea Institute of Science & Technology, Seoul, 02792, South Korea

[2] Korea University of Science and Technology, Daejeon, 34113, South Korea

[3] Advanced Analysis Center, Korea Institute of Science & Technology, Seoul, 02792, South Korea

[4] School of Advanced Materials Engineering, Kookmin University, Seoul, 02707, South Korea

***To whom correspondence should be addressed:**

Hojeong Jeon, Ph.D.

Senior Research Scientist, Center for Biomaterials, Biomedical Research Institute, KIST

Assistant professor for Korea University of Science and Technology

Tel : +82-2-958-5140, +82-10-9942-3516

Fax : +82-2-958-5449 E-mail : jeonhj@kist.re.kr

This PDF file includes:

Figs. S1 to S6, Table S1

Movies S1 to S2

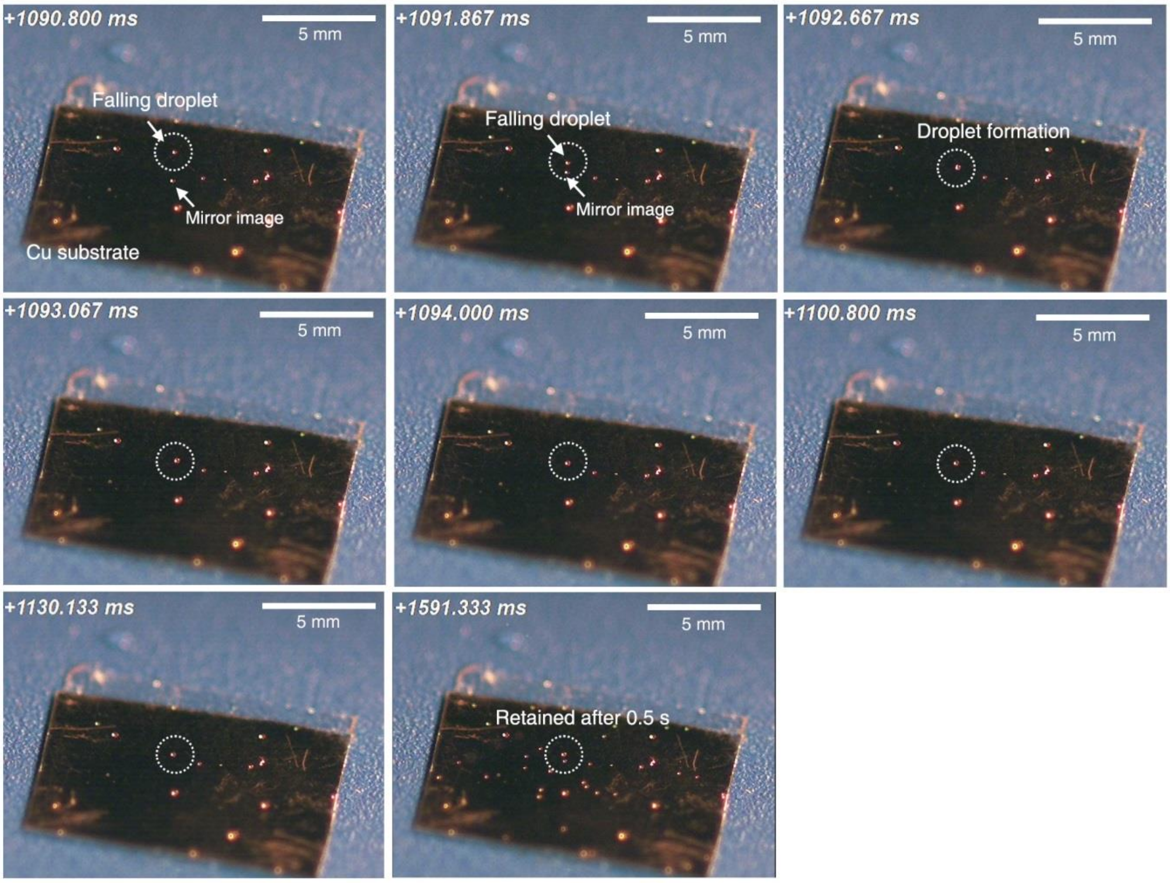

Figure S1. Snapshots of the sessile droplets before and after droplet formation. Although it is hard to predict the exact impact evolution of sessile droplets because of their small size, an apparent splash impact was not observed. The observed droplets retained their morphology after 0.5 s of droplet formation.

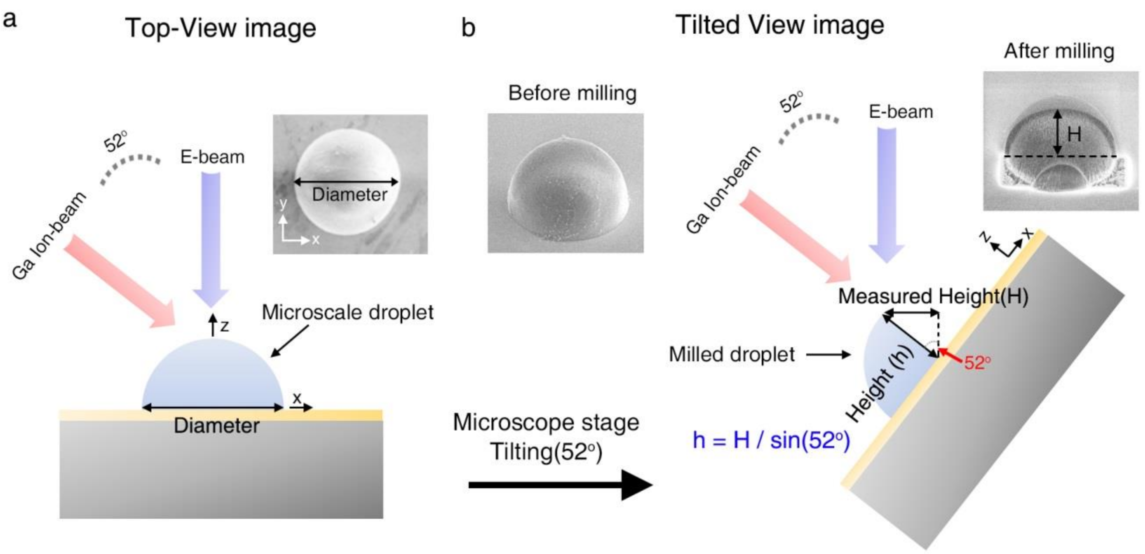

Figure S2. Schematics of the detailed contact angle measurement method. (**a**) The diameter of the droplets was measured using a top-view image before FIB milling. (**b**) After we tilted the microscope stage by 52° to make the z-axis of the droplets parallel to the ion-beam source, one-half of the droplets were removed with ion-beam milling. The measured height (H) was then corrected considering the angle between the z-axis of the droplet and electron-beam source, and the actual height (h) of the droplet was calculated.

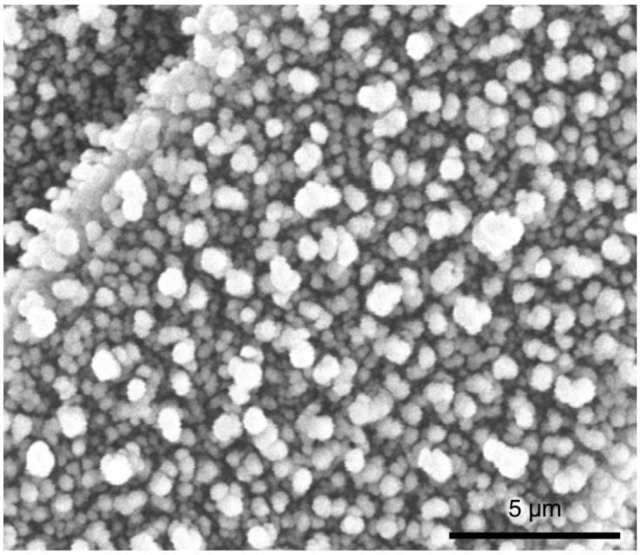

Figure S3. Tentatively formed granule-shaped ice particles on the substrates.

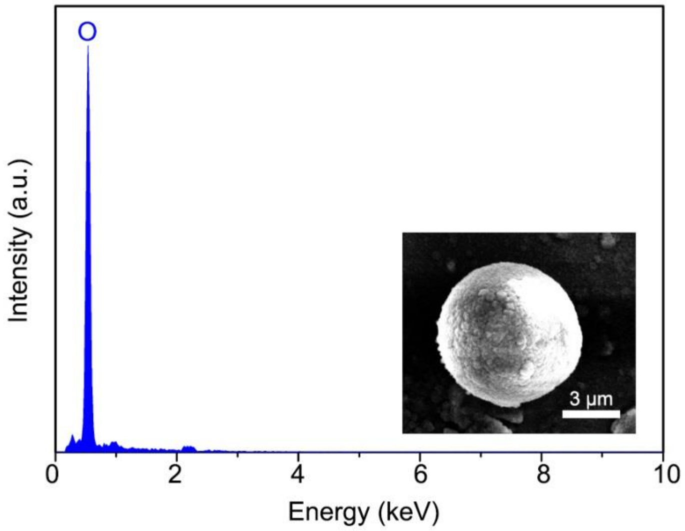

Figure S4. EDS spectra of an ultra-small microscale droplet. The EDS analysis showed that the droplet is mainly comprised of oxygen atoms, as was expected.

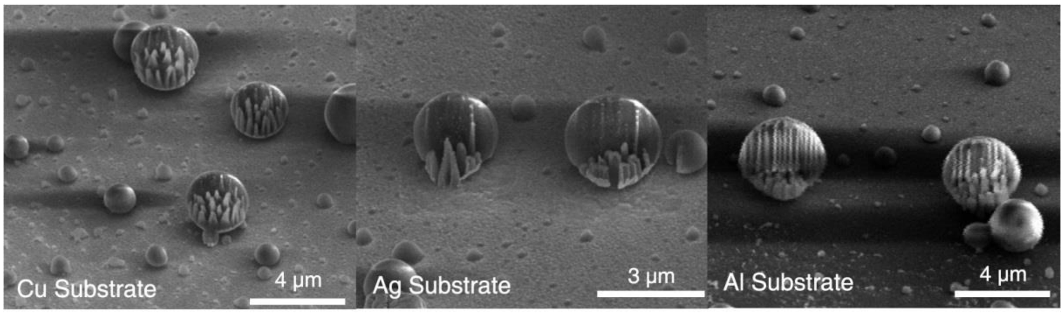

Figure S5. Spherical droplets on the Cu, Ag, and Al substrates. Cryo-SEM images of sub-5-μm diameter droplets on various metal substrates after FIB milling. The images were acquired by tilting the microscope stage to 52°.

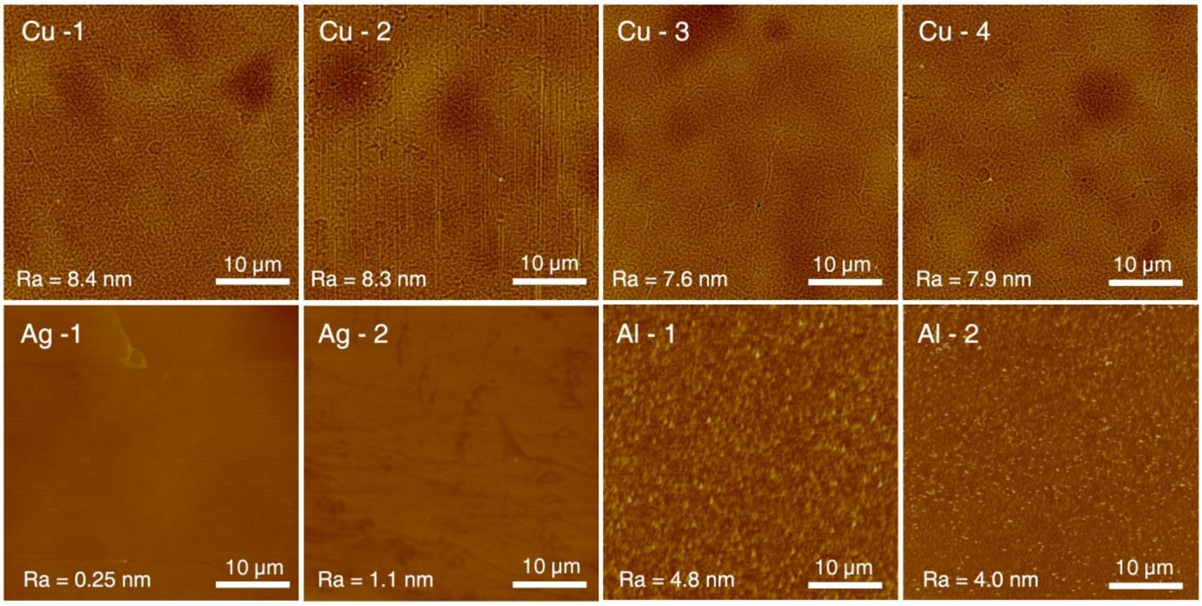

Figure S6. AFM images of various metal substrates. AFM images of independently prepared metal substrates with a detection area of 1,600 μm^2. The surface roughness value (R_a) of each substrate is indicated within the image.

Supplementary Table 1. Advancing contact angle, receding contact angle, and contact angle hysteresis of the droplets onto metal substrates measured by tilting method

Types	Advancing contact angle	Receding contact angle	Contact angle hysteresis
Cu	85.0 ± 3.8°	80.3 ± 3.6°	4.7 ± 1.7°
Ag	94.6 ± 4.4°	91.5 ± 4.8°	3.1 ± 1.3°
Al	56.4 ± 1.0°	54.0 ± 1.8°	2.4 ± 1.8°

Supplementary Movie Legend

Supplementary Movie 1.

A time-lapse image representing the formation pathway of sessile droplets on the Cu substrate recorded by high-speed camera.

Supplementary Movie 2.

A time-lapse image showing the sublimation of the spherical water droplets by raising the temperature of the metal stub on the microscope stage from -190 to 20℃.
